# Supplementary figures and images for: A Bayesian approach for estimating typhoid fever incidence from large‐scale facility‐based passive surveillance data
Source: Stat Med. 2021 Aug 24;40(26):5853–70. doi: 10.1002/sim.9159 (PMC9291985; doi:10.1002/sim.9159)

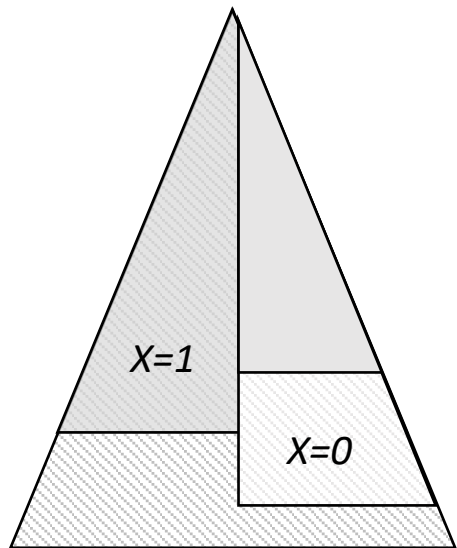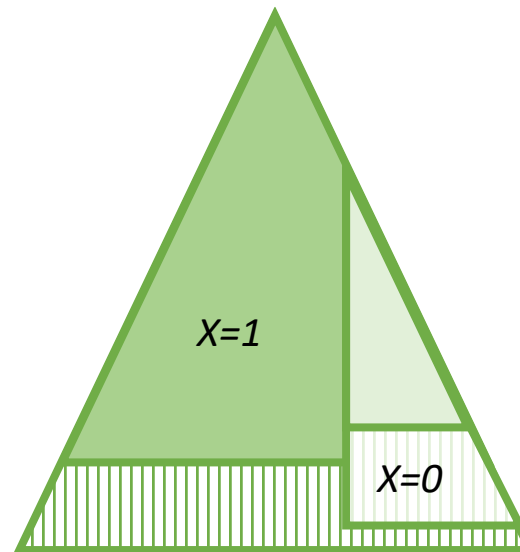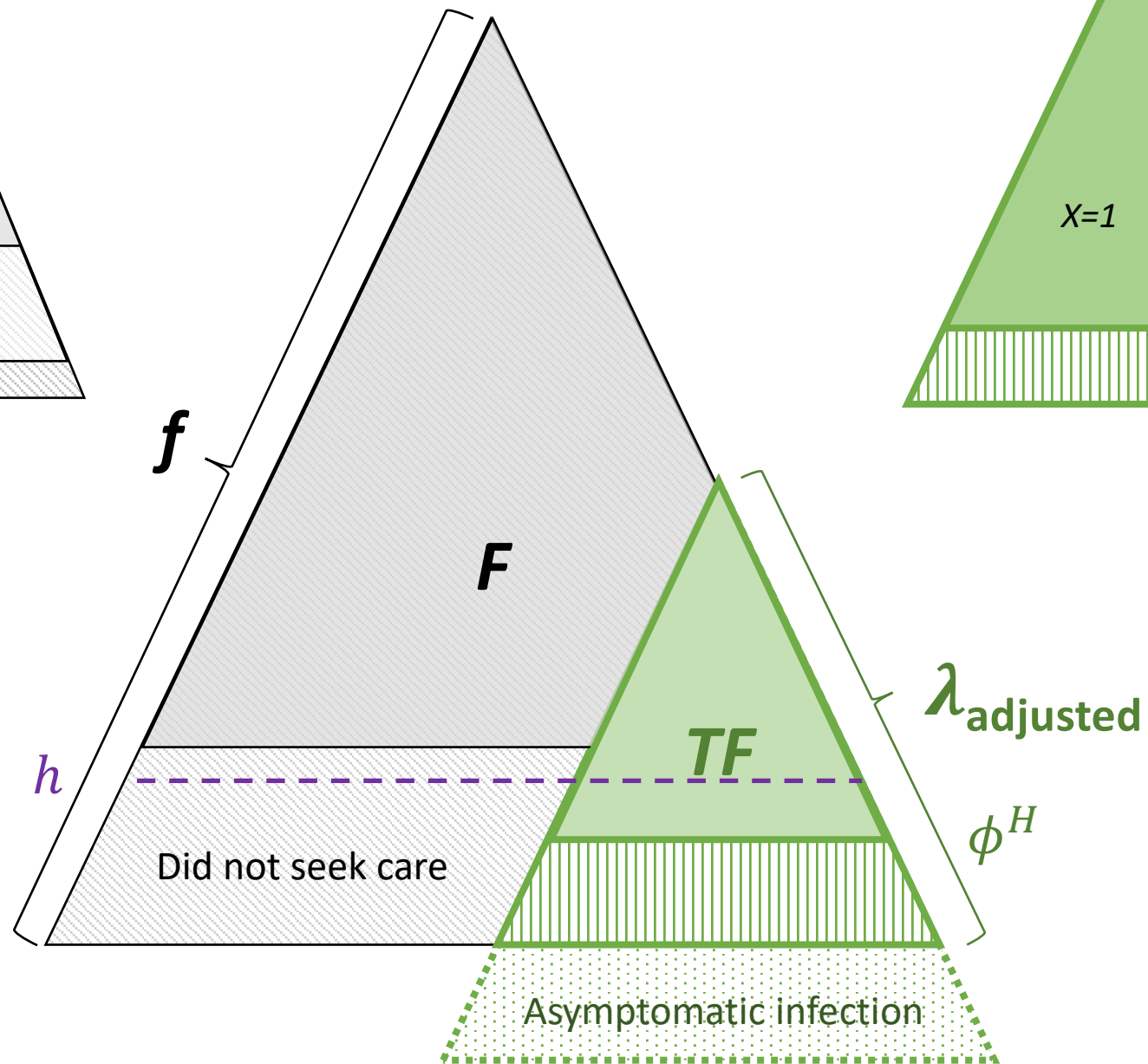

Supplement: Supplementary file 1 — Figure S1 Typhoid fever pyramid and febrile pyramid. The typhoid pyramid (green) is nested within the fever pyramid (gray). Some fraction of symptomatic typhoid fever cases and febrile cases seek care (shaded regions, TF and F, respectively). The average probability of seeking care for fever is measured (h; dashed purple line), but this may vary for individuals with typhoid fever vs fever due to other causes. Within the typhoid fever and fever pyramids, individuals may (X = 1) or may not (X = 0) have a risk factor for typhoid fever; the probability of seeking healthcare varies for those with or without the risk factor, and the risk factor is more prevalent among those with typhoid fever. One can observe whether a person has a fever, but not whether they have typhoid fever [file SIM-40-5853-s002.pdf]

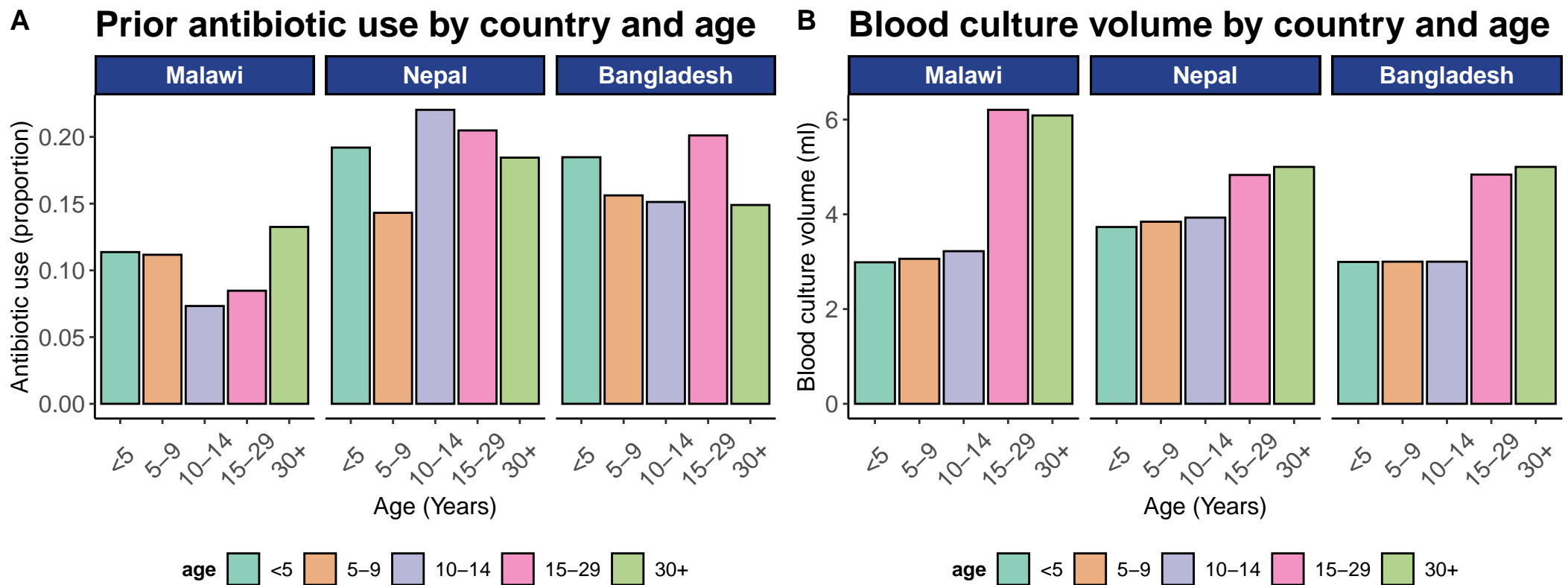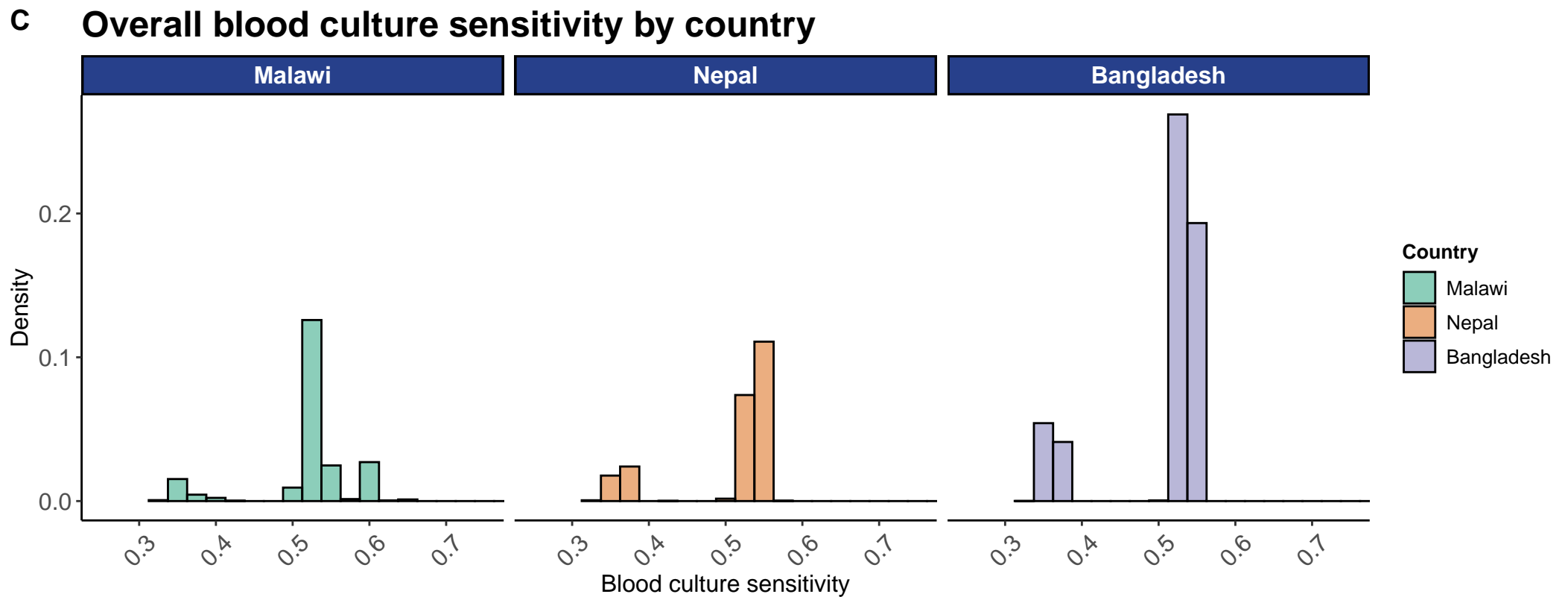

Supplement: Supplementary file 2 — Figure S2 Plots of prior antibiotic use, blood culture volume, and blood culture sensitivity. The average proportion of those with antibiotic use in the past 2 weeks, A and the average blood culture volume, B by country and age group is shown in plots A and B, respectively. In plot C, the distribution of overall (across all age groups) blood culture sensitivity after adjusting for prior antibiotic use and blood culture volume drawn is shown [file SIM-40-5853-s005.pdf]

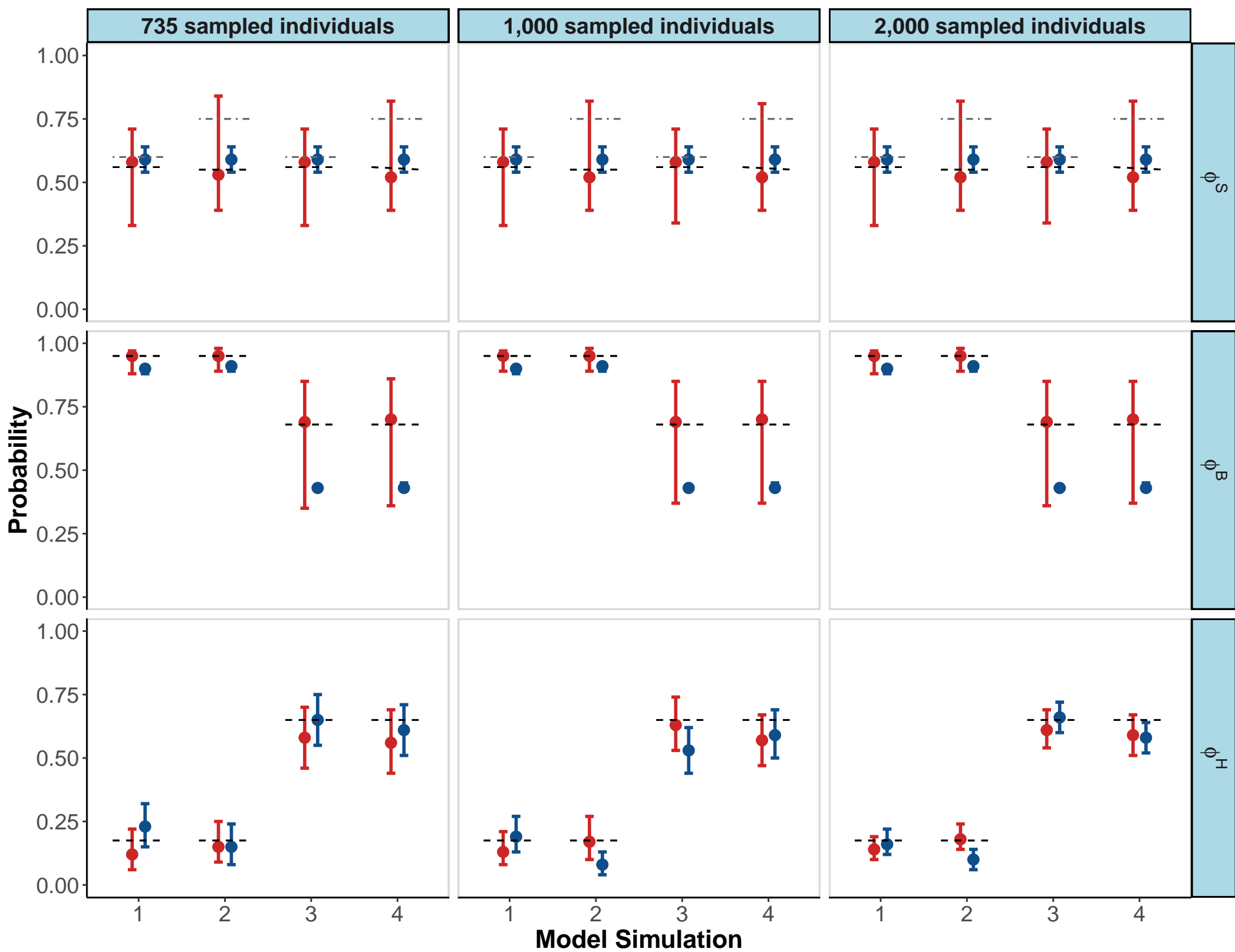

Supplement: Supplementary file 3 — Figure S3 Estimated probabilities from simulated data. Data for the estimated typhoid incidence were simulated for low, medium and high probabilities of seeking healthcare (ϕH), receiving a blood culture diagnostic test (ϕB), and blood culture sensitivity (ϕS) (row panels); and each simulation was performed sampling 735; 1000; and 2000 individuals (column panels) from the population to be “observed” from the healthcare utilization portion. The true values used for simulation are shown in dashed horizontal black lines. The value for blood culture sensitivity without adjusting for prior antibiotic use is shown in dotted gray lines. Estimated values are shown for models that did (red) and did not (blue) account for variation in blood culture sensitivity and variation in typhoid incidence among those who did or did not seek care and were or were not tested [file SIM-40-5853-s007.pdf]

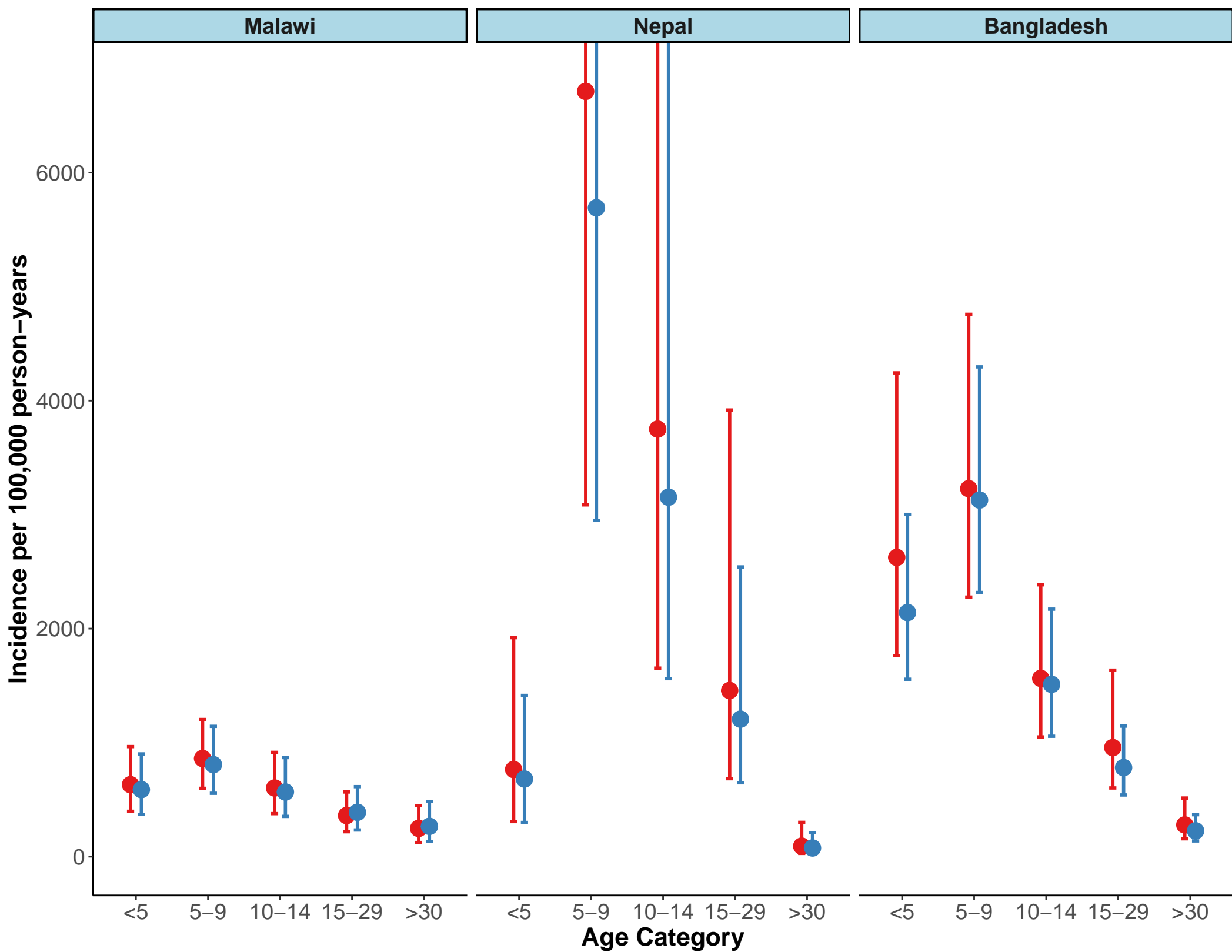

Supplement: Supplementary file 4 — Figure S4 Estimated STRATAA typhoid incidence with full model vs a simplified approach. The estimated typhoid incidence per 100 000 person‐years of observation is shown for models that did (red) and did not (blue) take into account variation in blood culture sensitivity and variation in typhoid incidence among those who did or did not seek care and were or were not tested for each age group and country. Note that the upper bounds on children 5 to 9 and 10 to 14 in Nepal are not shown [file SIM-40-5853-s004.pdf]

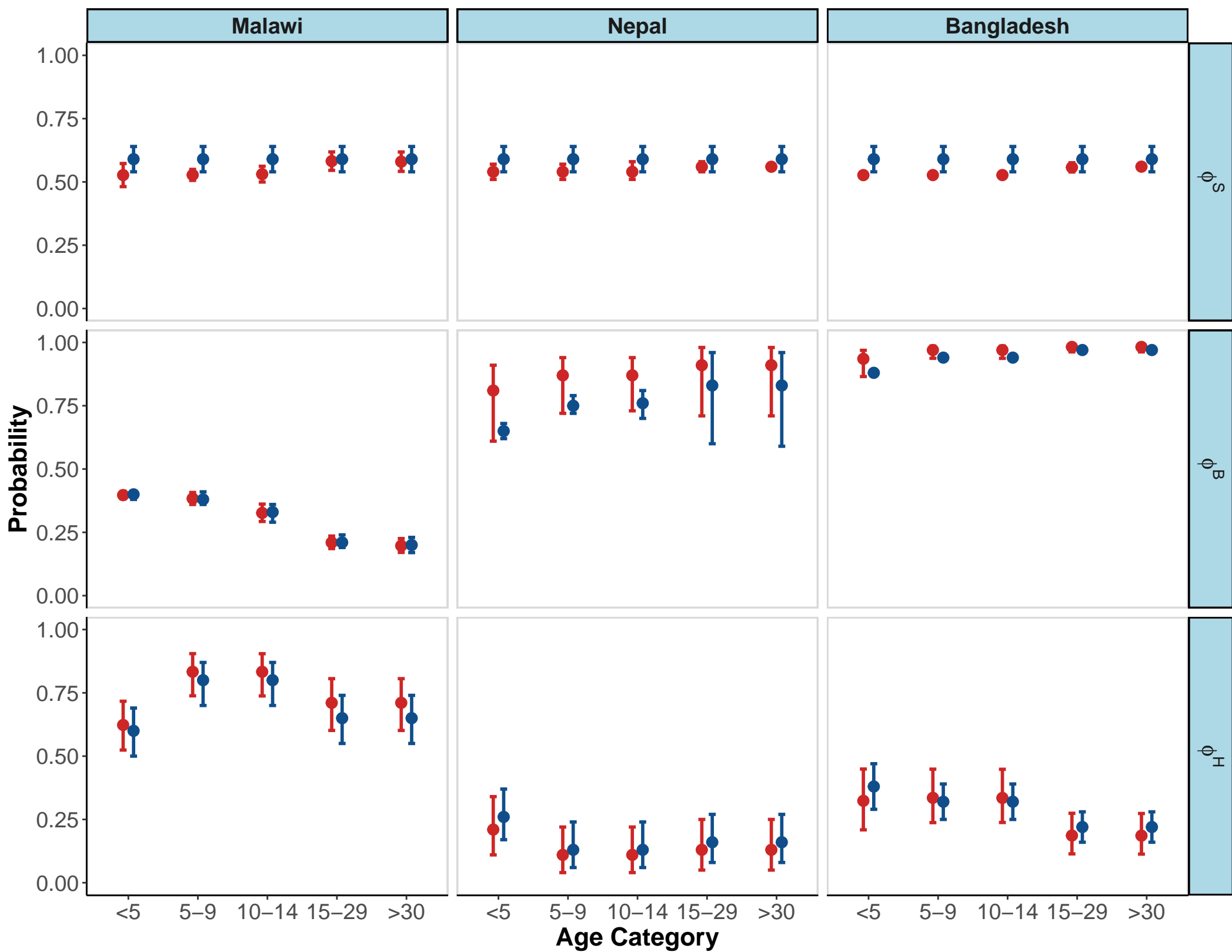

Supplement: Supplementary file 5 — Figure S5 Estimated STRATAA probabilities from full model vs a simplified approach. The estimated probabilities of seeking healthcare (ϕH), receiving a blood culture diagnostic test (ϕB), and blood culture sensitivity (ϕS) are shown for models that did (red) and did not (blue) take into account variation in blood culture sensitivity and variation in typhoid incidence among those who did or did not seek care and were or were not tested for each age group and country [file SIM-40-5853-s001.pdf]
