# Supplementary material for: A Bayesian approach for estimating typhoid fever incidence from large‐scale facility‐based passive surveillance data
Source: Stat Med. 2021 Aug 24;40(26):5853–70. doi: 10.1002/sim.9159 (PMC9291985; doi:10.1002/sim.9159)
Supplement: Supplementary file 6 — Table S1 Contingency table of an individual's sensitivity and specificity for blood culture diagnostic test. In this study, we assumed that all individuals who tested positive for typhoid fever were true cases of typhoid. Among those who tested negative, an individual i's probability of being a true case of typhoid (w i,v(i),u(i)) depended on the volume of blood drawn v and his or her reported prior antibiotic use u [file SIM-40-5853-s003.docx]

**Table S1. Contingency table of an individual’s sensitivity and specificity for blood culture diagnostic test.** In this study, we assumed that all individuals who tested positive for typhoid fever were true cases of typhoid. Among those who tested negative, an individual *i*’s probability of being a true case of typhoid (*w_i,v_*_(_*_i_*_)_*_,u_*_(_*_i_*_)_) depended on the volume of blood drawn *v* and his or her reported prior antibiotic use *u*.

|  | **Typhoid** | **No typhoid** |
| --- | --- | --- |
| **BC+** | *w_i,v_*_(_*_i_*_)_*_,u_*_(_*_i_*_)_ | 0 |
| **BC-** | 1 - w*_i,v_*_(_*_i_*_)_*_,u_*_(_*_i_*_)_ | 1 |
