# Supplementary material for: A Bayesian approach for estimating typhoid fever incidence from large‐scale facility‐based passive surveillance data
Source: Stat Med. 2021 Aug 24;40(26):5853–70. doi: 10.1002/sim.9159 (PMC9291985; doi:10.1002/sim.9159)
Supplement: Supplementary file 7 — Table S2 Prevalence of typhoid fever risk factor, rates of reported febrile illness, and probability of healthcare seeking from the Healthcare Utilization Surveys. The prevalence (prev.) and numerator used to calculate the prevalence (N) of each factor used to estimate the probability of healthcare seeking by age and country are show in the table below. Values are shown for the probability of having the risk factor for typhoid fever (P), and the proportion of those who sought care at a STRATAA partner health facility among those with (h 1) and without (h 0) the risk factor are shown [file SIM-40-5853-s006.docx]

**Table S2. Prevalence of typhoid fever risk factor, rates of reported febrile illness, and probability of healthcare seeking from the Healthcare Utilization Surveys.** The prevalence (prev.) and numerator used to calculate the prevalence (*N*) of each factor used to estimate the probability of healthcare seeking by age and country are show in the table below. Values are shown for the probability of having the risk factor for typhoid fever (*p*), and the proportion of those who sought care at a STRATAA partner health facility among those with (*h_1_*) and without (*h_0_*) the risk factor are shown.

|  |  | ***p*** | | ***h_1_*** | | ***h_0_*** | | ***R_TF_*** |
| --- | --- | --- | --- | --- | --- | --- | --- | --- |
|  | ***Age*** | ***prev.*** | ***N*** | ***prev.*** | ***N*** | ***prev.*** | ***N*** | ***Mean (95% CI)*** |
| **Nepal** | ***all ages*** | 0.42 | 130 | 0.13 | 11 | 0.25 | 27 | 5.7 (2.3-14.4) |
|  | ***u5*** | 0.37 | 28 | 0.18 | 5 | 0.32 | 15 |  |
|  | ***5-14*** | 0.45 | 58 | 0.10 | 3 | 0.19 | 5 |  |
|  | ***15+*** | 0.42 | 44 | 0.12 | 3 | 0.20 | 7 |  |
| **Bangladesh** | ***all ages*** | 0.30 | 863 | 0.26 | 35 | 0.31 | 95 | 7.6 (2.2-26.5) |
|  | ***u5*** | 0.29 | 146 | 0.29 | 10 | 0.41 | 31 |  |
|  | ***5-14*** | 0.30 | 306 | 0.35 | 16 | 0.31 | 34 |  |
|  | ***15+*** | 0.30 | 411 | 0.17 | 9 | 0.24 | 30 |  |
| **Malawi** | ***all ages*** | 0.20 | 378 | 0.74 | 59 | 0.64 | 130 | 2.0 (1.3-2.5) |
|  | ***u5*** | 0.20 | 126 | 0.72 | 23 | 0.55 | 42 |  |
|  | ***5-14*** | 0.20 | 126 | 0.78 | 18 | 0.80 | 47 |  |
|  | ***15+*** | 0.20 | 126 | 0.72 | 18 | 0.62 | 41 |  |
